# The DNA methylation landscape of naturally short-lived killifish

**DOI:** 10.1038/s41598-026-39352-3

**Published:** 2026-02-19

**Authors:** Mara Steiger, Nishita Singh, Alexandra M. Tyers, Helene Kretzmer, Dario Riccardo Valenzano, Alexander Meissner

**Affiliations:** 1 Max Planck Institute for Molecular Genetics, Berlin, Germany; 2 Digital Engineering Faculty, Hasso Plattner Institute for Digital Engineering, University of Potsdam, Potsdam, Germany; 3 Max Planck Institute for Biology of Ageing, Cologne, Germany; 4 Leibniz Institute on Aging - Fritz Lipmann Institute (FLI), Jena, Germany; 5 Institute of Chemistry and Biochemistry, Freie Universität Berlin, Berlin, Germany

**Keywords:** Ecology, Ecology, Evolution, Genetics, Zoology

## Abstract

Annual killifish of the genus *Nothobranchius* are naturally short-lived vertebrates that exhibit a wide range of aging characteristics typically observed in mammals. Here, we provide the first comprehensive whole-genome methylation map for two species, revealing local age-related as well as tissue-specific dynamics in the DNA methylome. Our maps and results support the annual killifish as a model organism and further highlight its value for studying aging including possible health status predictions in vertebrates.

**Supplementary Information:**

The online version contains supplementary material available at 10.1038/s41598-026-39352-3.

## Introduction

DNA methylation is an important epigenetic mechanism that has been linked to aging and age-related diseases^1–3^. The addition of a methyl group to cytosine residues in DNA (most prevalent in the CpG context, i.e., cytosine followed by guanine) can alter genome regulation without changing the underlying DNA sequence. Across vertebrates, aging is accompanied by characteristic and largely conserved changes in DNA methylation. These are small-level global drifts in DNA methylation patterns, including hypomethylation of CpGs in repetitive sequences and hypermethylation of focal regions^4,5^, such as promoters and cis-regulatory elements. Together, these changes form the basis of mammalian “epigenetic clocks,” which use a subset of these age-associated CpG sites to robustly predict chronological and biological age, as well as correlate with mortality risk and age-related physiological decline^6,7^. Although these methylation hallmarks are well established in humans and other mammals^8^, it remains unclear whether similarly conserved aging signatures exist in naturally short-lived, non-mammalian vertebrates such as annual killifish, or whether extreme lifespan compression alters epigenetic aging dynamics.

Annual killifish of the genus *Nothobranchius* are a powerful vertebrate system to address this question. Species in this genus are small freshwater fish that have evolved an exceptionally rapid life cycle and a specialized life strategy to cope with their periodically extreme environment. Females lay eggs in small pools of water that dry up quickly after the rainy season. The eggs enter a state of diapause, a developmental arrest that allows them to survive in the dry environment until the next rainy season. During the next rainy season, the hatchlings mature quickly and reach sexual maturity within a few weeks^9–12^, after which they have a brief reproductive lifespan. With a median lifespan of 3–6 months, *N. furzeri* is one of the shortest-lived vertebrates known^13–15^. In comparison, its close relative *N*. *orthonotus* lives substantially longer (median lifespan of up to 10 months^16^) despite occupying a nearly identical ecological niche. This is in stark contrast to similarly sized animals (3–6 cm), such as the zebrafish *Danio rerio*, which lives up to 5.5 years (median lifespan 3 years^17^), or the smallest mammal, the Etruscan shrew *Suncus etruscus* (maximum/median lifespan 3.2^18^/1.3^19^ years, respectively). Both *Nothobranchius* species display spontaneous onset of dramatic phenotypic changes as they age, including decreased fertility, changes in swimming ability and learning capacity^20^, alterations in skin and eye pigmentation^12,13,21,22^, loss of antibody diversity^23^, intestinal dysbiosis^24^, neurodegeneration^20,25^, and the spontaneous onset of neoplasias^26,27^. These characteristics make annual killifish, specifically those of the genus *Nothobranchius*, an attractive model for investigating the molecular and genetic mechanisms underlying aging and for developing interventions to extend healthy lifespan.

Despite growing use of annual killifish in aging biology, little is known about their epigenetic genome regulation by DNA methylation, as no genome-wide maps exist yet. Prior targeted analyses in *N. furzeri* revealed age-related reductions in the expression of DNA methylation maintenance enzymes and in global methylation content (5mC), with variation across tissues, as measured by dot blot^28^. Based on these findings, we hypothesized that age-related DNA methylation in killifish is associated with genome-wide loss and with localized hypermethylation at gene-regulatory elements in a tissue-specific manner. This underscores the need for base-resolution methylation maps to characterize the extent and tissue-type specificity of these changes. Establishing such reference maps is relevant for determining whether short-lived vertebrates display the canonical aging-associated methylation signatures, whether lifespan differences between closely related species correlate with distinct epigenetic trajectories, and whether annual killifish methylomes can support the development of age-predictive tools analogous to those found in mammalian epigenetic clocks^6,7^.

Here, we generate the first whole-genome bisulfite sequencing maps for two *Nothobranchius* species across tissues and ages. We characterize their baseline methylation architecture, identify tissue-specific and age-associated methylation changes, and compare aging trajectories between the short-lived *N. furzeri* and the longer-lived *N. orthonotus*. These data provide a foundational resource for annual killifish epigenomics and offer insight into the conservation and divergence of aging-associated DNA methylation patterns in vertebrates.

## Results

**Fig. 1:**
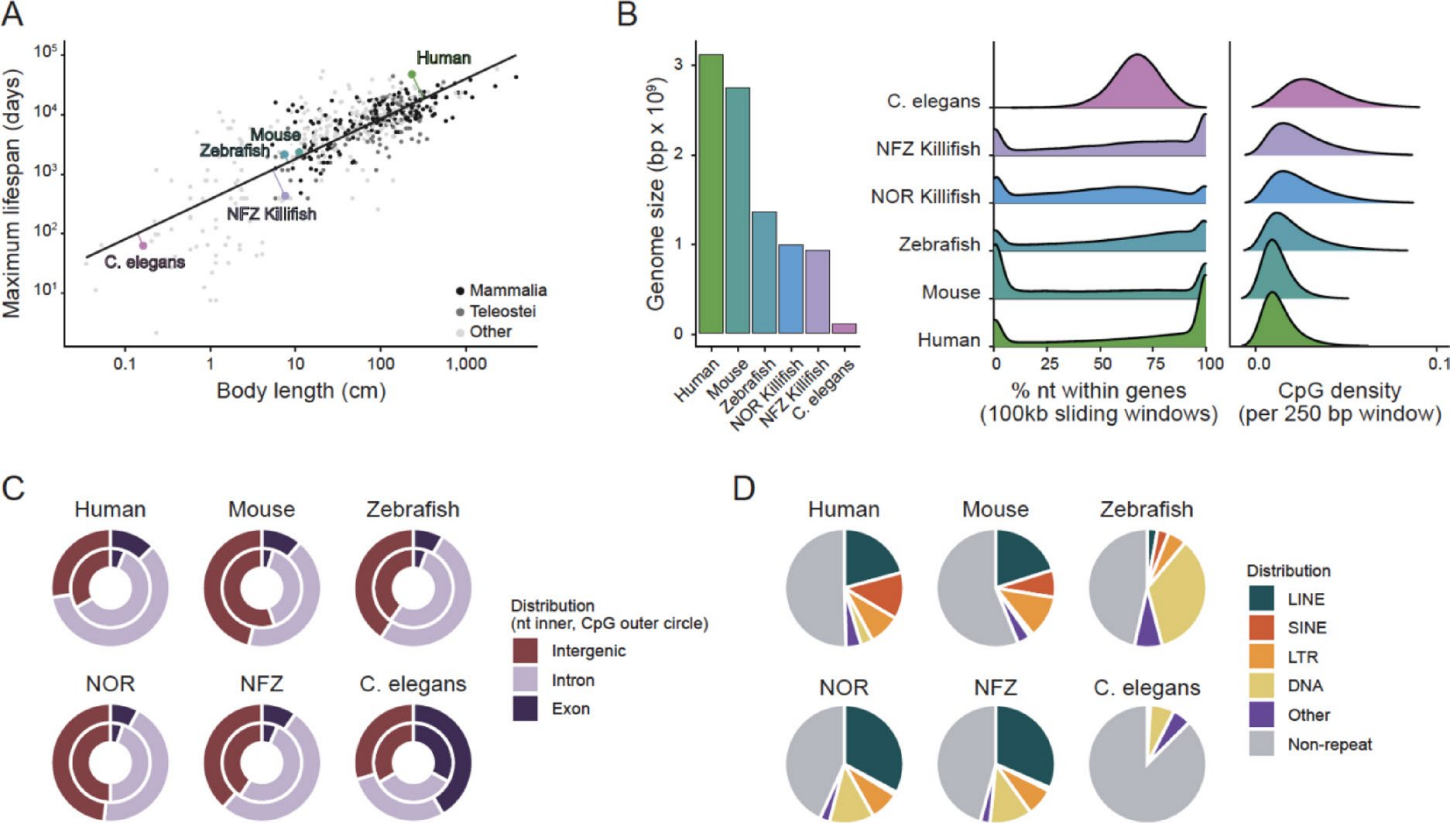
Genomic landscape of *Nothobranchius furzeri* (NFZ) and *Nothobranchius orthonotus* (NOR) compared to human, mouse, zebrafish, and C. elegans. (**A**) Body length (in cm) vs. maximum lifespan (in days) across species in the GTDrift database, shown on a log–log scale. Each point represents a species; mammalian species are shown in black, teleost fish in grey, and all other species in light grey. The black line indicates a linear regression across all species. Selected species are labeled, and their deviations from the regression line are highlighted. (**B**) Comparison of genomic features of different model organisms. (left) Species’ total genome length (in bp × 10^9) and (right) Density plots of gene density (left panel) and CpG density (right panel) across sliding windows. Genome assemblies used are NFZv2.0 for *N. furzeri*, NORv1p2 for *N. orthonotus*, hg38 for human, mm10 for mouse, danRer11 for zebrafish, and ce11 for *C. elegans*. (**C**) Genomic feature composition across exons, introns, and intergenic regions (inner ring: fraction of nucleotides; outer ring: fraction of CpGs within respective features). (**D**) Genomic repeat composition showing proportion of LINEs, SINEs, LTRs, DNA repeats, other repetitive sequences, and non-repetitive regions.

Lifespan usually scales with body size across vertebrates and, to some degree, in non-vertebrates. However, annual *Nothobranchius* killifish species deviate strongly from this pattern, living only a few months despite an adult body length comparable to small mammals surviving one to two years (among the top 3 shortest-lived mammals and teleostei (253 species); based on relative residuals; Fig. 1A). To evaluate if this is reflected in a markedly different genome structure, we first compared the genomic architecture of *N. furzeri* (NFZ) and *N. orthonotus* (NOR) with longer-lived vertebrates of different lifespans (human, mouse, zebrafish), as well as the short-lived invertebrate *C. elegans*. *C. elegans* here reflects an outgroup to the other analyzed species, since it lacks canonical 5mC methylation in the CpG sequence context^29^ and therefore represents a genomic architecture not shaped by methylation-associated CpG depletion. Despite similar genome sizes (~ 1.5 Gb for *N. furzeri*^30^ and *N. orthonotus*^31^) to zebrafish (~ 1.4 Gb^32^), both annual killifish species exhibit notably shorter lifespans, with *N. furzeri* reaching a maximum lifespan of less than 1 year compared to ~ 5.5 years in zebrafish. Although genome size has occasionally been discussed in relation to lifespan in fish and other taxa^33^, more comprehensive analyses across vertebrates have found no consistent relationship^34,35^. Even within annual killifish, *N. orthonotus* lives on average ~ 50% longer than *N. furzeri* despite nearly identical genome sizes (Fig. 1B, left). Gene density and CpG distribution in both annual killifish are similar to zebrafish, while mammals show greater compartmentalization of gene- and CpG-rich regions (Fig. 1B, right). CpG enrichment across genomic features deviated significantly from genomic background (χ^2^ test, *p* < 1e-304), and standardized residuals confirmed enrichment of CpGs in exonic regions, with stronger enrichment in mammals and moderate deviations in *N. furzeri* and *N. orthonotus*. In the genomes of annual killifish and zebrafish, CpGs are slightly enriched in coding regions (exons: ~ 5–6% of genome, ~ 8–10% of CpGs, Fig. 1C), while mammals exhibit a more pronounced CpG enrichment in genic regions, with exons accounting for ~ 5–6% of the genome but contributing ~ 11–13% of CpGs. In contrast, *C. elegans* shows a peak at 50–75% genic content reflecting its compact genome organization with minimal intergenic space (Fig. 1B, right). This is consistent with the more uniform genome-wide CpG distribution and the relatively even allocation of CpGs across exonic and intronic regions (Fig. 1B, C).

In all vertebrates, approximately half of the genome is composed of repetitive elements (Fig. 1D). In annual killifish, LINEs and DNA repeats are the predominant classes. While lacking the abundant SINE elements found in human and mouse genomes, they share elevated LINE content. Despite their phylogenetic proximity to zebrafish, the repeat architecture of annual killifish more closely resembles that of mammals. The zebrafish repeat landscape, in contrast, is dominated by DNA transposons, and LINEs are nearly absent. *C. elegans* has minimal overall repetitive content and a composition primarily made up of DNA repeats.

Taken together, these comparative analyses show that the markedly shorter lifespan of annual killifish cannot be explained by gross genomic features such as genome size, gene density, or CpG organization. The absence of any obvious genomic feature that explains their shorter lifespans motivated us to explore epigenetic regulation, specifically DNA methylation dynamics, as a potential contributor to their rapid aging trajectories.

**Fig. 2:**
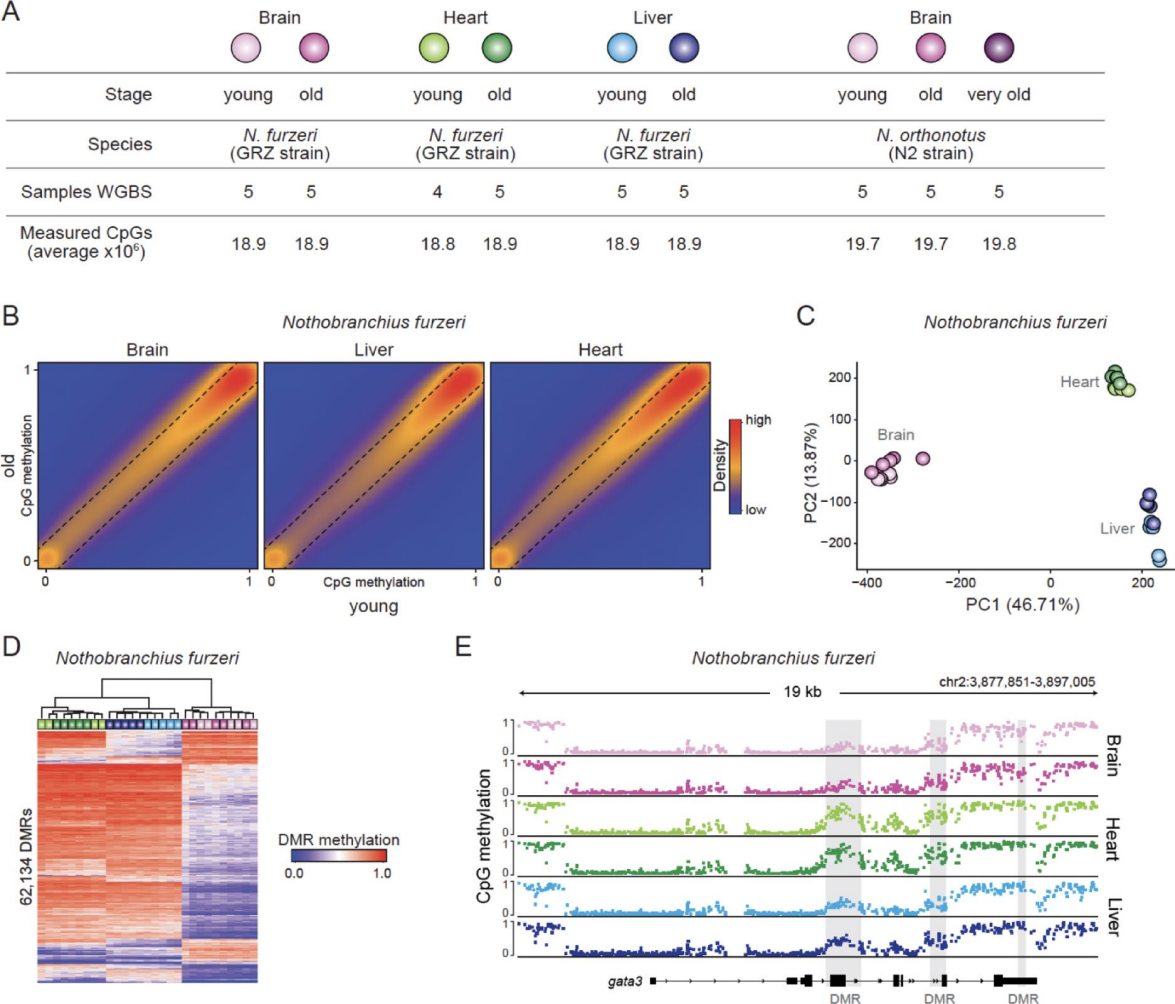
The killifish methylome is bimodally distributed and exhibits tissue-specific dynamics. (**A**) Description of the samples used in this study. (**B**) Comparison of mean CpG methylation levels between young and old brain, liver, and heart tissue samples from *N. furzeri*. N = 18,764,787 CpGs. (**C**) Principal component analysis (PCA) of all *N. furzeri* samples used in this study (based on 17,083,785 CpGs). (**D**) Heatmap representation of the methylation levels of 62,134 differentially methylated regions (DMRs) found between the samples from the three tissues of *N. furzeri*. (**E**) Browser shot of mean CpG methylation levels highlighting differentially hypomethylated regions in brain compared to liver and heart in *N. furzeri* samples.

To establish a solid reference for DNA methylation in annual killifish, we generated high-coverage whole-genome bisulfite sequencing (WGBS) data of individual-matched brain, liver, and heart tissue from young and old *N. furzeri* (GRZ strain; ~ 46 and ~ 110 days, respectively) as well as brain tissue from young, old, and very old *N. orthonotus* (N2 strain, ~ 44, ~ 112, and ~ 263 days, respectively; very old only reached in captivity) (Fig. 2A, Supplementary Table 1). We sequenced 20 billion fragments (150nt paired-end), resulting in an average of 18.6 million CpGs with a mean coverage of 50X across the 19 autosomes (Supplementary Table 1). On a genome-wide scale, the annual killifish methylation landscape exhibits a conserved bimodal distribution, with approximately 75% of CpGs being highly methylated and about 10% showing very low methylation rates, displaying little variability between individuals and conditions (Supplementary Fig. 1A–C). Similar to mammalian genomes, transcription start sites tend to be CpG-rich and less methylated than the gene body and intragenic regions, with little to no differences between tissues on this scale (Supplementary Fig. 1D).

Focusing initially on our *N. furzeri* data set covering different tissues and ages, we find highly correlated methylation patterns within a tissue between young and old individuals (Fig. 2B). In line with this observation, the primary source of variance between individual samples seems to be tissue rather than age, as indicated by variance decomposition via linear models fitted to the first 10 PCs ((tissue) adj. *p*-value < 0.05 for PC1 and PC2, (age) adj. *p*-value < 0.05 for PC2 and PC4, Fig. 2C, Supplementary Fig. 2A, B). In concordance with germ layer specification during early embryonic development, the CpG methylation landscapes in heart and liver samples, originating from meso- and endoderm, are more similar to each other than they are to brain (ectoderm). To quantify the level, frequency, and distribution of methylation differences between the three tissues, we called differentially methylated regions (DMRs^36^) between all pairwise combinations (Supplementary Table2). While liver vs heart showed fewer than 10k DMRs, about five times as many DMRs were found between liver and heart compared to brain (Supplementary Fig. 2C). The majority of these DMRs were hypomethylated, indicating that selected regions are consistently less methylated across samples in a brain-specific manner (Fig. 2D). These include some well-known neuronal marker genes like *gata3*, *ncam1*, *neurod1,* and members of the Sox family, suggesting potential regulatory relevance of these regions (Fig. 2E, Supplementary Fig. 2D, E).

Beyond genome-wide methylation differences between tissues, principal component analysis on the *N. furzeri* methylome suggested that age contributes to additional explained variance among samples (Fig. 2C, Supplementary Fig. 2A, B). Notably, a recent comparative study of annual and non-annual killifish species found that annual killifish have undergone genome expansion under relaxed selection. This expansion was driven largely by transposable element (TE) proliferation and accompanied by elevated mutation loads in aging-related genes, suggesting an association with their rapid aging and markedly shortened lifespans^31^. Given the prominence of transposable elements in annual killifish genomes and emerging evidence that age-related TE derepression contributes to genomic instability and aging phenotypes in other species/organisms^37–39^, we next investigated methylation dynamics specifically at transposable elements.

**Fig. 3:**
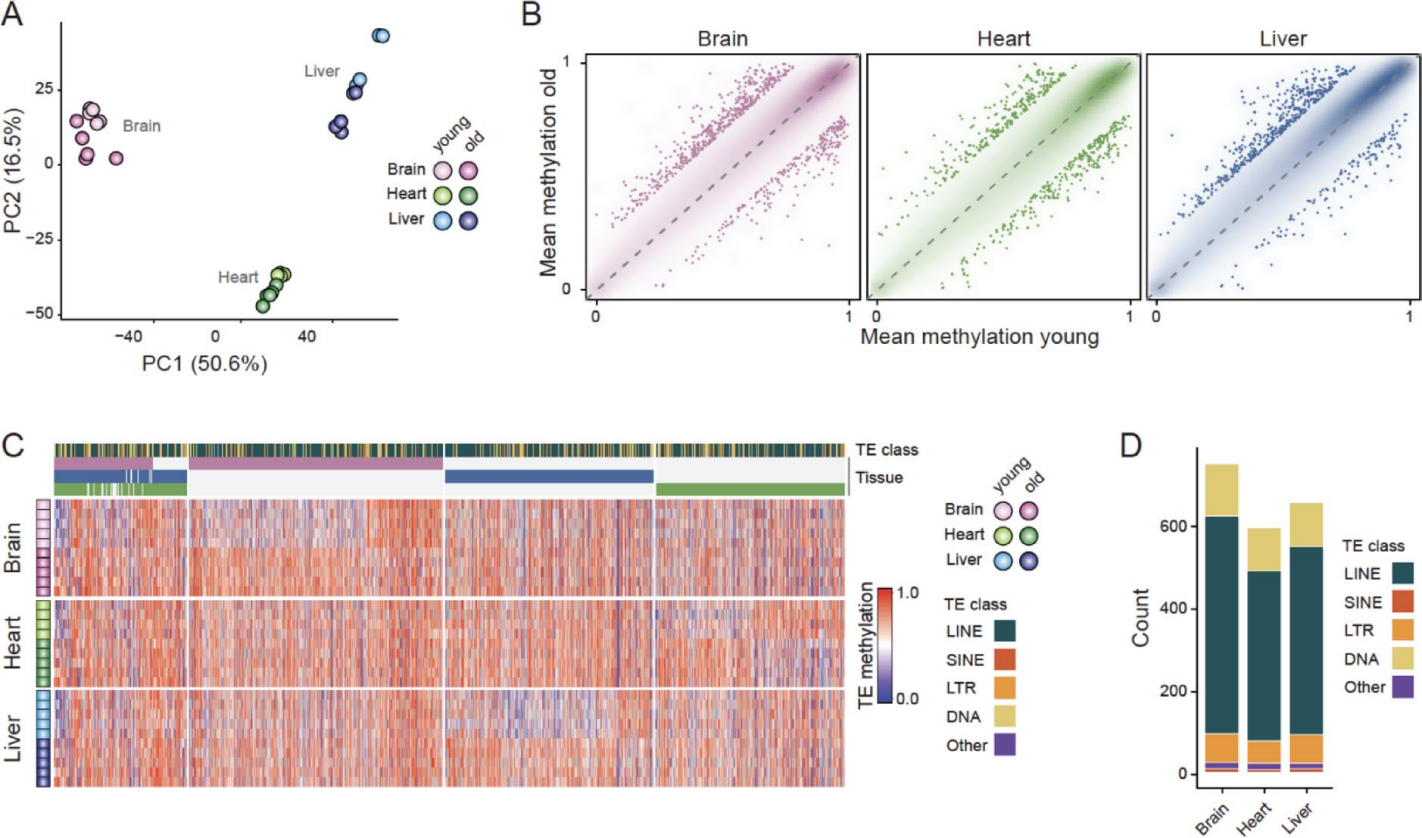
NFZ shows global maintenance of TE methylation with locus-specific remodeling during aging. (**A**) PCA of all *N. furzeri* samples used in this study (based on mean methylation of N = 1,343,612 TEs). (**B**) Comparison of mean TE methylation between young and old brain (left), heart (middle), and liver (right) samples from *N. furzeri* (N = 1,384,504, N = 1,386,253, N = 1,385,799 TEs, respectively). Smooth scatterplot shows genome-wide distribution, individual TEs with absolute methylation differences > 0.2 are overlaid as points (N = 747, N = 592, N = 652 TEs, respectively). (**C**) Heatmap representation of the methylation levels for N = 1,656 differentially methylated TEs across all brain, heart, and liver samples of *N. furzeri*. Column annotations indicate TE class and tissue-specific differential methylation. (**D**) Counts of differentially methylated TEs per tissue, grouped by TE class.

PCA of average methylation of TEs revealed similar clustering by tissue and, to some degree, by age group, as seen with genome-wide CpGs (variance decomposition via linear models fitted to the first 10 PCs: (tissue) adj. *p*-value < 0.05 for PC1 and PC2, (age) adj. *p*-value < 0.05 for PC1, PC2, and PC4, Figs. 2C, 3A, Supplementary Fig. 3A,B. Despite lower dimensionality of the data, this might suggest that age-related changes at repeats are slightly more pronounced than global methylation shifts. At the same time, mean TE methylation showed generally stable distributions between young and old samples across classes, consistent with the global methylation stability observed for individual CpGs (Fig. 3B, Supplementary Fig. 3C; compare with Fig. 2A). To identify elements potentially driving this separation, we called differentially methylated TEs in each tissue (delta methylation >= 0.2, min. covered by at least two samples). We detected 747 age-associated TEs in brain (240 hypo-, and 507 hypermethylated), 652 in liver (144 hypo-, and 508 hypermethylated), and 592 in heart (295 hypo-, and 297 hypermethylated) (Fig. 3B,C, Supplementary Table 3). Consistent with their abundance in the *N. furzeri* genome, differentially methylated TEs were predominantly LINE TEs (log2 fold change < 0.1) and showed a minor enrichment in DNA or SINE repeat families depending on the tissue (Fig. 3D, Supplementary Fig. 3D). However, no TE class was significantly enriched or depleted in differentially methylated TEs relative to the genomic background (Fisher’s exact test: adj. *p*-values > 0.05). Despite tissue-specific variation, we identified a shared subset of 56 TEs with consistent age-associated methylation changes across all three tissues, and an additional 223 TEs that were shared between two tissues (Fig. 3C). Gene ontology analysis of genes located near differentially methylated TEs did not yield clear functional enrichment (Supplementary Fig. 3E), suggesting that TE hyper- and hypo-methylation with age is widespread rather than targeted to specific biological pathways/gene networks.

**Fig. 4:**
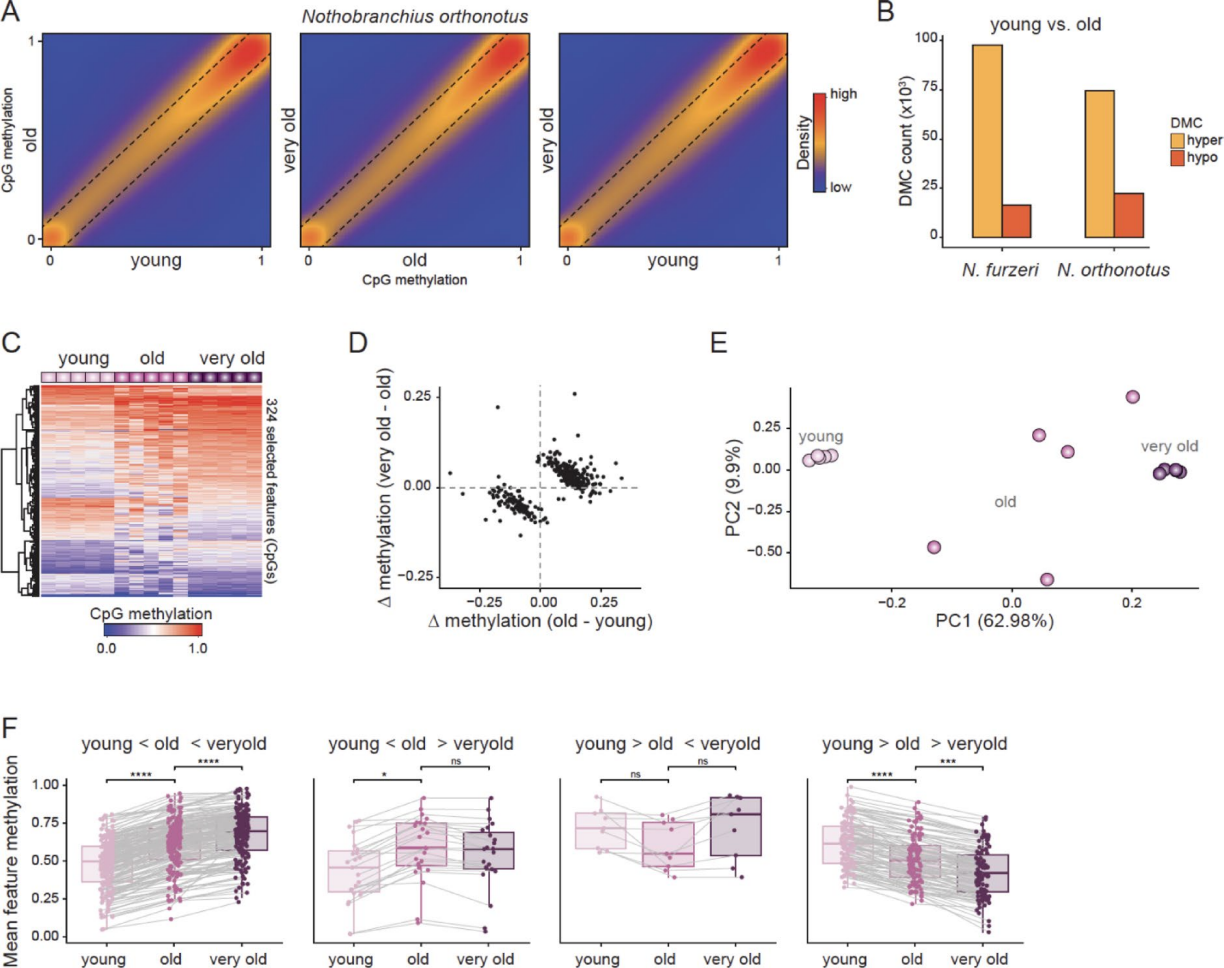
CpG methylation differences indicate an aging fingerprint on the methylome. (**A**) Comparison of mean CpG methylation levels between young, old, and very old samples of brain tissues from *N. orthonotus*. N = 19,402,262 CpGs. (**B**) Number of hypo- and hypermethylated DMCs between young and old samples in brain tissues. (**C**) Heatmap representation of the methylation levels of N = 324 selected features from the linear model on young and very old *N. orthonotus* brain samples. (**D**) Comparison of mean methylation differences between young and old, as well as old and very old brain *N. orthonotus* samples in these selected features. (**E**) PCA of all *N. orthonotus* brain samples used in this study based on these selected features. (**F**) Mean methylation dynamics in these selected features. Left to right: N = 186, 23, 9, 106 features. In the boxplot, the centerline is median; boxes, first and third quartiles; whiskers, 1.5 × inter-quartile range; additionally, all data is displayed as points. Significance levels from unpaired Wilcoxon tests are indicated (*p ≤ 0.05, p** ≤ 0.01, p*** ≤ 0.001, p**** ≤ 0.0001; ns, not significant).

Given that targeted analyses revealed localized, age-associated methylation dynamics, especially at transposable elements, despite largely stable global and TE methylation levels in *N. furzeri*, we next asked whether these focal changes are a function of time or lifespan. Here, we focused on brain samples, which were available from the short-lived *N. furzeri* and long-lived *N. orthonotus*. Similar to *N. furzeri* brain tissue, no strong genome-wide alterations between age groups were detected in *N. orthonotus* (Figs. 2B, 4A). In line with this, the absolute number of differentially methylated CpGs (DMCs) between young and old is comparable in *N. furzeri* and *N. orthonotus* (Fig. 4B, Supplementary Table 4). Interestingly, the CpGs that differ between young and old samples tend to rather bounce back to young methylation levels in the very old samples than deviate further. This trend has recently been reported for mice^40^, and was also found when training a multinomial regression model on the data (elastic net regularization resulting in 61 age group-predictive features for the age groups, Supplementary Fig. 4, Supplementary Table 5). Since the brain exhibits a heterogeneous cell-type composition, we hypothesize that nonlinear methylation changes may reflect this heterogeneity. To mitigate this effect, we next trained a linear regression model on the young and very old samples, resulting in 324 age-predictive features (log-transformed age in days, Fig. 4C-F, Supplementary Table 5). Most of these showed continuous changes across all groups, separating the young from the very old samples, with the old samples more similar to the very old.

## Discussion

We provide the first genome-wide maps of two annual killifish species, *Nothobranchius furzeri* and *Nothobranchius orthonotus*, and describe their canonical methylation landscapes and tissue-specific dynamics. A slight but detectable methylation change between age groups suggests that the annual killifish epigenome, similar to other organisms, undergoes age-dependent changes over the individuals’ life span, highlighting its potential for age-predictive tools that are frequently used in mammals^41–43^. These observations are in alignment with patterns described in mammals (localized shifts at regulatory/repetitive regions with limited global change), but the effect sizes are small. It is tempting to speculate that a compressed lifespan might limit cumulative drift. The lack of strong pathway enrichment near age-responsive elements is also consistent with distributed, low-amplitude change rather than targeted pathway regulation. Of note, our cross-species comparison did not allow firm conclusions about whether the longer-lived *N. orthonotus* ages more slowly at the methylome level. Inference is constrained by small sample sizes, discretized ages, analysis of a cell-type heterogeneous tissue (brain), and remaining dependence on current assemblies and annotations.

Despite these limitations, an important outcome is the establishment of a reference resource with baseline methylation architecture across tissues, candidate age-responsive CpGs and regions, and TE loci with age-associated changes. Building precise epigenetic clocks for annual killifish will require substantially larger, more diverse cohorts with denser age sampling and independent validation. Overall, the data support the view that age-related methylation changes are present but limited in these short-lived vertebrates and are shaped by species-specific genomic context.

## Methods

### Annual killifish samples

Breeding, embryo collection, hatching, and fish husbandry followed standard protocols^44^. Fish were reared in the fish facility at the Max Planck Institute for Biology of Ageing and euthanized by anesthetic overdose (600 mg/L MS222 in tank water) administered by trained personnel. The following LANUV permits applied: Holding license acc. §11TSchG (license number 576.1.36.6.G12/18) and Announcement acc. §4 TSchG for preparation of tissue (license number: MPIa_Anzeige§4_RB.16.005). Fish were dissected to extract the brain, liver, and heart, which were immediately flash-frozen in liquid nitrogen and stored at − 80˚C until use. Tissues from *N. furzeri* were collected at ~ 46 and ~ 110 days (young and old), and from *N. orthonotus* at ~ 44, ~ 112, and ~ 263 days (young, old, and very old) post-hatching (see Supplementary Table 1 for individual ages).

All annual killifish procedures were carried out in accordance with German animal welfare legislation. Fish were euthanized for organ collection under LANUV permits (Holding license acc. §11 TSchG: 576.1.36.6.G12/18; Announcement acc. §4 TSchG: MPIa_Anzeige§4_RB.16.005). No experimental interventions were performed on live animals. The study is reported in accordance with ARRIVE guidelines, where applicable.

### Whole-genome bisulfite sequencing (WGBS)

Bisulfite converted DNA libraries were prepared using the Accel-NGS Methyl-Seq DNA library kit (SWIFT BIOSCIENCES). In brief, 200 ng (in 50 µl) of purified DNA was fragmented to ~ 350 bp using the Covaris S2 system (10% duty cycle, intensity 5 for 2 × 45 s in Covaris AFA tubes) followed by a concentration step with Zymo columns (DNA Clean & Concentrator). 20 µl of DNA were used for overnight bisulfite conversion with the Zymo EZ-DNA methylation Gold Kit. Bisulfite converted DNA was fully denatured by 2 min incubation at 95 °C and immediately transferred to ice. To anneal a truncated adapter, 15 µl of denatured DNA was mixed with 25 µl of Adaptase reaction mix (SWIFT) and incubated for 15 min at 37 °C, 2 min at 95 °C, and then cooled to 4 °C. Extension and second strand synthesis by using a primer complementary to the truncated adapter was performed by mixing the adaptase reaction mix with 44 µl of extension reaction mix (SWIFT) and incubation at 98 °C for 1 min, 62 °C for 2 min, 65 °C for 5 min, and cooling to 4 °C. The samples were cleaned with 1.2 volumes of Ampure XP beads, 80% ethanol, and eluted in 15 µl. Ligation of the second (truncated) adapter was performed at 25 °C for 15 min followed by an additional bead cleanup with 1 volume of AmpureXP beads and 80% ethanol. Samples were individually indexed using unique dual indexed primer sets with 6 cycles of a slightly modified PCR program (30 s @ 98 °C, 6 cycles of 15 s @ 98 °C, 30 s @ 60 °C, 60 s @ 68 °C, followed by a final 5 min incubation step at 68 °C. Libraries were finally cleaned up with 1 volume of Ampure XP beads. Quality was assessed using Agilent’s Bioanalyzer, and concentration was determined in qPCR. Libraries were pooled equimolarly and sequenced on an Illumina NovaSeq 6000 or HiSeq4000 machine with paired-end 150 mode.

### Genome comparison

Genome size estimates were compiled from calculations based on sequencing depth for annual killifish genomes^30,45^ and published near-complete assemblies for human^46^, mouse^47^, zebrafish^32^, and *C. elegans* (WBcel235). Annotations of body length and maximum lifespan for all species shown were retrieved from the GTDrift database^18^. *Galeopterus variegatus* was excluded because the lifespan entry in GTDrift sourced via EOL^48^ strongly deviated from alternative literature^49^. Genome assemblies used for all other comparative analyses were NFZv2.0 for *N. furzeri,* NORv1p2 for *N. orthonotus,* hg38 for human, mm10 for mouse, danRer11 for zebrafish, and ce11 for *C. elegans.* Gene annotations were retrieved from ENSEMBL release 114 (zebrafish and *C. elegans*), GENCODE Release 48 (human), and GENCODE Release M25 (mouse). Repeat annotations for human, mouse, zebrafish, and *C. elegans* were obtained from RepeatMasker tracks via the UCSC table browser^50^. Gene density was calculated as the percentage of nucleotides overlapping with annotated genic regions (exons and introns) using a sliding window approach (100 kb window size, 20 kb step size). CpG density was calculated as the fraction of CpG dinucleotides within 250 bp windows using a sliding window approach (50 bp step size). All analyses were restricted to canonical chromosomes, excluding chrM as well as unplaced scaffolds and contigs.

### WGBS processing

Raw reads were subjected to adapter and quality trimming using cutadapt (v2.4; parameters: –quality-cutoff 20–overlap 5–minimum-length 25; Illumina TruSeq adapter clipped from both reads), followed by trimming of 10 and 5 nucleotides from the 5’ and 3’ end of the first read and 15 and 5 nucleotides from the 5’ and 3’ end of the second read^51^. The trimmed reads were aligned to the *Nothobranchius furzeri* and *Nothobranchius orthonotus* reference genomes (NFZv2.0 for *N. furzeri,* NORv1p2 for *N. orthonotus*) using BSMAP^52^ (v2.90; parameters: –v 0.1 –s 16 –q 20 –w 100-S 1-u-R). A sorted BAM file was obtained and indexed using samtools^53^ with the ‘sort’ and ‘index’ commands (v1.10). Methylation rates were called for autosomes only, using mcall from the MOABS^54^ package (v1.3.2; default parameters). All analyses were restricted to autosomes, and only CpGs covered by at least 10 and at most 200 reads were considered for downstream analyses. Assessment of global, genome-wide methylation, as well as region-specific methylation, was performed using average (arithmetic mean) methylation levels. Replicates were grouped after filtering for coverage by calculating the arithmetic mean of the remaining positions given their presence in at least one replicate.

### Differential methylation analysis

Differentially methylated regions (DMRs) were called using metilene^36^ (v0.2–7). DMRs were defined to have an absolute minimum difference in methylation of 0.3 with a maximum distance of 300nt between CpGs within a DMR and a minimum of 10 CpGs per DMR and Bonferroni correction for multiple testing (parameter –c 1 –d 0.3). DMRs were calculated between all *N. furzeri* (i) brain and liver (ii) brain and heart, and (iii) liver and heart samples.

Differentially methylated CpGs (DMCs) were called using metilene (v0.2–7). DMCs were defined to have an absolute minimum difference in methylation of 0.1 in DMC mode (parameter -f 3). DMCs were calculated between (i) young and old *N. furzeri* brain samples and (ii) young and old *N. orthonotus* brain samples.

### TE methylation

A species-specific repeat annotation for *N. furzeri* was generated using RepeatMasker (v4.1.9) with an input library of TEs obtained from FishTEDB^55^. The tool was run in sensitive mode (-s) to improve detection of low-copy repeats, with the -nolow flag to exclude low-complexity sequences, and the -no_is flag to disable detection of bacterial insertion sequences. Mean methylation of TEs was assessed by calculating average methylation levels using bigWigAverageOverBed from UCSC tools for each sample or group, where a feature was only considered if at least three CpGs were covered within a region. Differentially methylated TEs were identified per tissue by selecting TEs with a minimum difference of 0.2 in mean methylation between young and old samples, with group means computed from at least two samples. TEs were annotated with the nearest gene using the rtracklayer R package^56^, with a maximum distance of 10 kb. GO enrichment was performed using the gost function from the grprofiler2 R package^57^ (organism = “nfurzeri”), with FDR applied for multiple testing correction.

### Elastic net age prediction models

CpG sites were filtered for i) missing values and ii) variance > 0.0075 across the respective samples resulting in 2,354,286 CpG sites as independent variables for the multinomial logistic regression model. Age groups as dependent variable were regressed on the CpG methylation rates with elastic net regularization using the glmnet^58^ package in R. A mixing parameter of α = 0.9 was chosen and an optimal regularization intensity λ was identified by tenfold cross-validation. With respect to the observation of local non-continuous methylation dynamics across age groups in *N. orthonotus* (Fig. 4C, D)*,* a second model was fitted on young and very old brain samples only. Here, log-transformed age in days was regressed on CpG methylation rates using a linear regression model with elastic net regularization (α = 0.2).

### Data analysis and visualization

Genome-browser visualizations were performed in IGV^59^ (v2.14.1) and gene-profile plots using deeptools^60^. All further analysis and visualization were performed in R^61^ (v4.2.2). Genome-wide correlations of CpG methylation and mean TE methylation between age groups were plotted using the mean methylation rate across samples per condition using SmoothScatter. All PCAs were calculated using the prcomp package. Contributions of covariates to variations in the principal components were analyzed by fitting linear models to scores of the first ten PCs. To quantify variance explained by each covariate, partial R^2^ values were computed and evaluated using F-tests with Benjamini–Hochberg correction. For heatmap visualization, the ComplexHeatmap^62^ and circlize^63^ packages were used. Bar plots, scatter plots, box plots, donut plots, density plots, pie charts, and PCAs were visualized using ggplot2^64^. Pairwise differences between respective means in boxplots were tested for significance using unpaired Wilcoxon tests. Violin plots were generated using the vioplot package and show the kernel density estimation with embedded boxplots indicating the median, interquartile range, and whiskers extending to 1.5 × the interquartile range.

## Supplementary Information

Below is the link to the electronic supplementary material.

Supplementary Material 1

Supplementary Material 2

Supplementary Material 3

Supplementary Material 4

Supplementary Material 5

Supplementary Material 6

## Data Availability

All sequencing data have been deposited in the Gene Expression Omnibus (GEO) under accession code GSE225702. Processed methylation data is publicly available via Zenodo (10.5281/zenodo.18401352).

## Code availability

All computational code used in this study is publicly available via Zenodo (10.5281/zenodo.18414629).
