# Supplementary material for: The DNA methylation landscape of naturally short-lived killifish
Source: Sci Rep. 2026 Feb 19;16:7173. doi: 10.1038/s41598-026-39352-3 (PMC12921331; doi:10.1038/s41598-026-39352-3)
Supplement: Supplementary file 1 — Supplementary Material 1 [file 41598_2026_39352_MOESM1_ESM.docx]

# Supplementary Figures


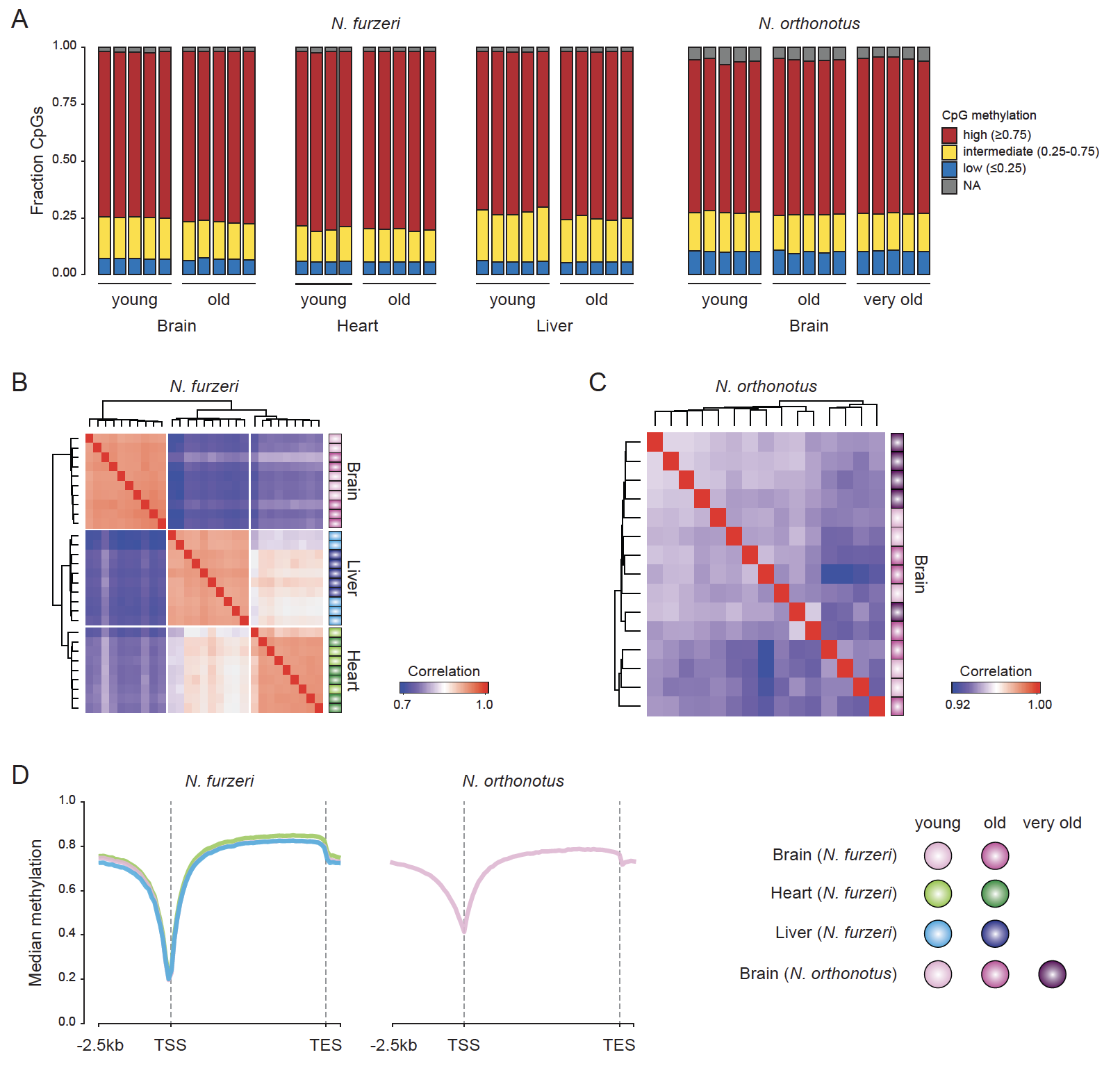


**Supplementary Fig. 1: Global features of the Killifish DNA methylome.** A) Fraction of low, intermediate, and highly methylated CpGs per sample. Number of CpGs covered 19,047,058 and 20,012,986 in N. furzeri and N. orthonotus samples. B) Genome-wide methylation rate correlation heatmap between all N. furzeri samples. Based on 17,913,747 CpGs covered by all samples. C) Genome-wide methylation rate correlation heatmap between all N. orthonotus samples. Based on 17,083,785 CpGs covered by all samples. D) Average methylation profiles across genes for N. furzeri and N. orthonotus genomes.


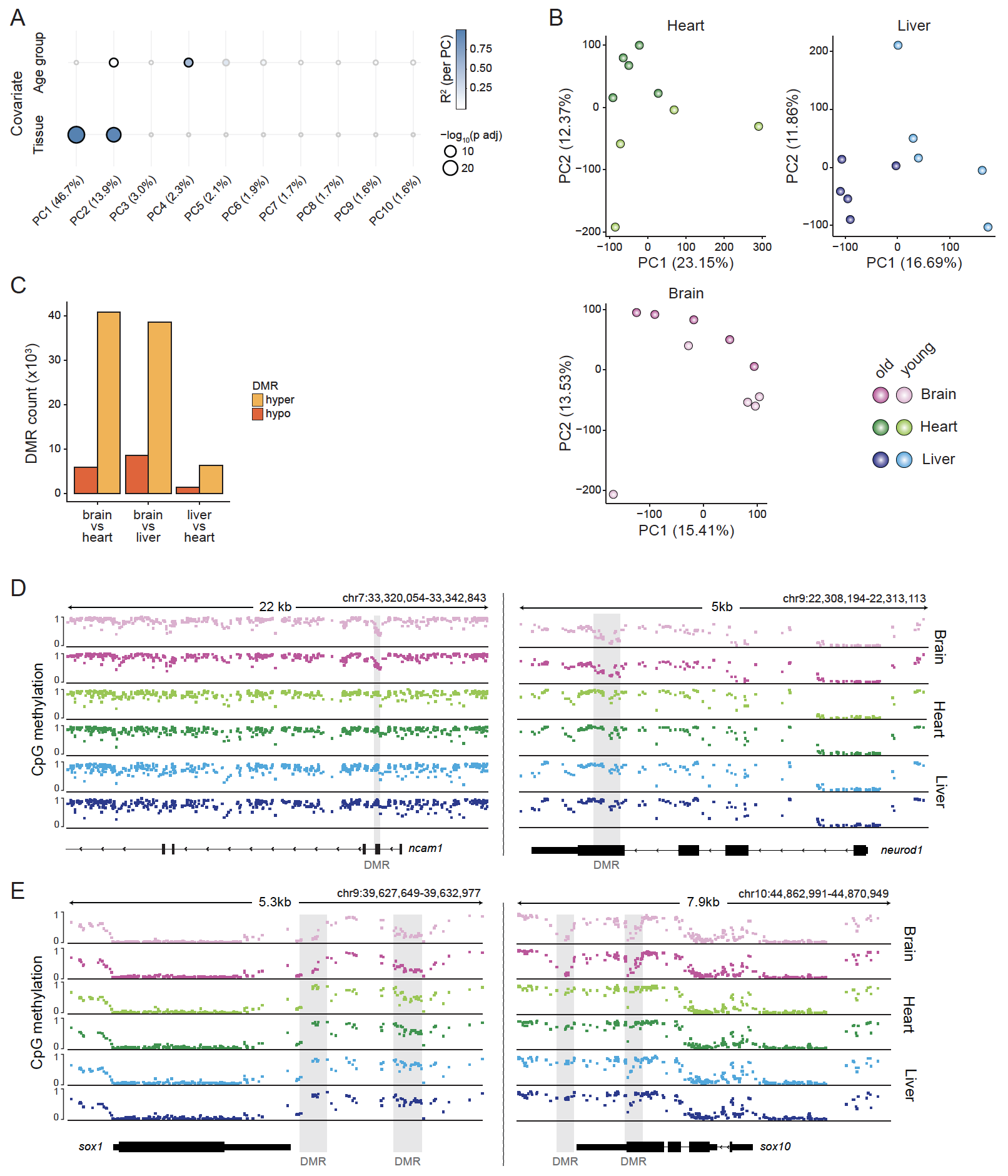


**Supplementary Fig. 2: Methylation differences separate N. furzeri samples by tissue.** A) Visualization of the contribution of age and tissue to the variance explained by the first ten PCs using linear modeling with the covariates as predictors. Circle color indicates explained variance (partial R^2^) and size indicates -log_10_-transformed adjusted F-test p-values (<0.05: black outline). B) Individual PCA of all N. furzeri samples used in this study. N = 17,913,747 CpGs covered by all samples. C) Number of DMRs detected between brain, heart, and liver N. furzeri samples. D) Fold-change of differentially methylated repeat elements vs. global distribution (log_2_). Other includes all families not specifically named, see Supplementary Table 3. E) Browser shots of mean CpG methylation levels highlighting differentially hypomethylated regions in brain compared to liver and heart in N. furzeri samples.


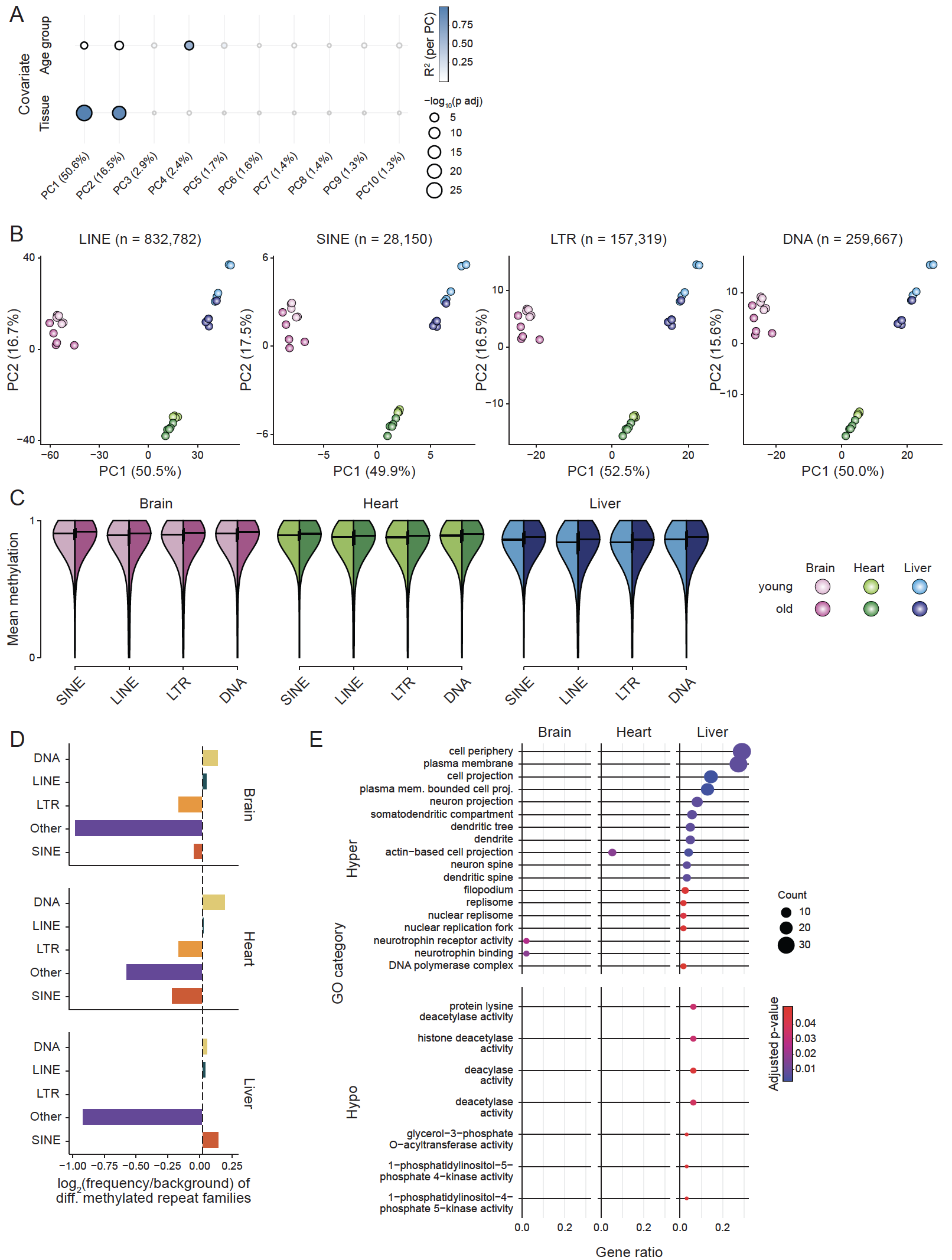


**Supplementary Fig. 3: Methylation of transposable elements across tissues and age groups in N. furzeri. A)** Visualization of the contribution of age and tissue to the variance explained by the first ten PCs using linear modeling with the covariates as predictors. Circle color indicates explained variance (partial R^2^) and size indicates -log_10_-transformed adjusted F-test p-values (<0.05: black outline). B) PCA of all N. furzeri samples used in this study based on mean methylation of N = 832,782 LINEs, N = 28,150 SINEs, N = 157,319 LTRs, and N = 259,667 DNA repeats. C) Distribution of average methylation levels for major TE classes (LINE, SINE, LTR, DNA) in young and old samples, shown separately by tissue. D) Log2-fold enrichment of differentially methylated TEs in each repeat class, relative to their frequency in the N. furzeri genome. E) Gene ontology (GO) analysis of nearest genes located within 10kb of differentially hyper- and hypo-methylated TEs between young and old brain, liver, and heart methylation profiles.


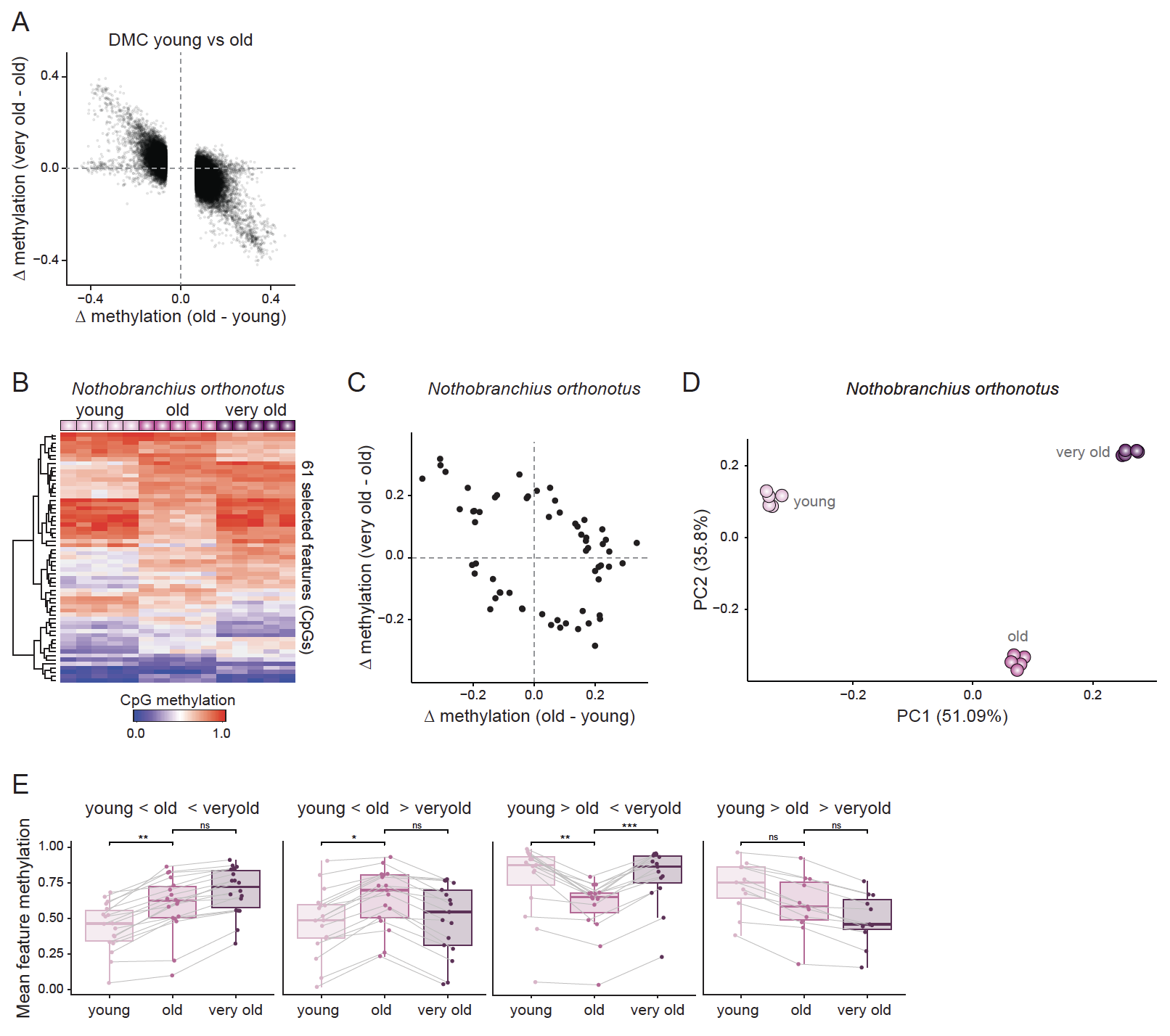


**Supplementary Fig. 4: Methylation dynamics across age groups in brain samples.** A) Comparison of methylation differences between young and old as well as old and very old brain samples in DMCs between young and old N. orthonotus samples. B) Heatmap representation of the methylation levels of the N = 61 selected features from multinomial model on young, old, and very old N. orthonotus brain samples. C) Comparison of mean methylation differences between young and old as well as old and very old N. orthonotus brain samples in these selected features. D) PCA of all N. orthonotus brain samples used in this study based on these selected features. E) Mean methylation dynamics in N = 61 selected features from multinomial regression model across age groups separated by continuity and direction. Left to right: N = 18, N = 17, N = 15, N = 11 features. In the boxplot, the centerline is median; boxes, first and third quartiles; whiskers, 1.5 x inter-quartile range; additionally, all data is displayed as points. Significance levels from unpaired Wilcoxon tests are indicated (*P ≤ 0.05, P** ≤ 0.01, P*** ≤ 0.001; P**** ≤ 0.0001; ns, not significant).
